# A zona incerta-basomedial amygdala circuit modulates aversive expectation in emotional stress-induced aversive learning deficits

**DOI:** 10.3389/fncel.2022.910699

**Published:** 2022-08-26

**Authors:** Lijun Zhang, Pei Zhang, Guangjian Qi, Hongwei Cai, Tongxia Li, Ming Li, Chi Cui, Jie Lei, Kun Ren, Jian Yang, Jie Ming, Bo Tian

**Affiliations:** ^1^Department of Neurobiology, School of Basic Medicine, Tongji Medical College, Huazhong University of Science and Technology, Wuhan, China; ^2^Institute for Brain Research, Huazhong University of Science and Technology, Wuhan, China; ^3^Key Laboratory of Neurological Diseases, Ministry of Education, Wuhan, China; ^4^Department of Breast and Thyroid Surgery, Union Hospital, Medical College, Huazhong University of Science and Technology, Wuhan, China

**Keywords:** emotional stress, aversive expectation, zona incerta, basomedial amygdala, TH^+^ circuit, vicarious social defeat stress

## Abstract

A previously published study showed that stress may interfere with associative aversive learning and facilitate mood-related disorders. However, whether emotional stress alone affects aversive learning is unknown. Using three chamber-vicarious social defeat stress (3C-VSDS) model mice, we investigated the effect of emotional stress on aversive learning. An important origin of dopamine (DA) neurons, the zona incerta (ZI), is expected to be a novel target for the modulation of aversive learning. However, less is known about the circuit mechanism of ZI*^DA^* neurons in aversive learning. Here, we subjected mice to a fear-conditioning system (FCS) and observed an increased calcium activity of ZI TH^+^ neurons in aversive expectation during the conditioning phase, especially during the late stage of the conditional stimulus (CS) when CS and unconditional stimulus (US) pairings were used. Optogenetic inhibition of ZI TH^+^ neurons at the late stage of CS disrupted conditioned fear learning in mice. We further identified a TH^+^ projection from the ZI to the basomedial amygdala (BMA) and found that optogenetic inhibition of the ZI-BMA circuit could also block aversive learning. Finally, we used 3C-VSDS mice as a model of emotional stress. We found that the 3C-VSDS model mice demonstrated reduced aversive expectation associated with ZI TH^+^ neurons in the late stage of CS and impaired aversive learning in FCS. Optogenetic activation of ZI-BMA TH^+^ projections in the late stage of CS significantly reversed the aversive FCS learning disability of 3C-VSDS model mice. These data suggest that a TH^+^ circuit from the ZI to the BMA is required for aversive expectation, both at baseline and in 3C-VSDS-induced aversive learning deficits and that this circuit is a potential target for the modulation of aversive learning. Low activity of ZI-BMA TH^+^ projections is one reason for 3C-VSDS-induced aversive learning deficits.

## Introduction

Exposure to emotional stress, acute or chronic, is a generally acknowledged factor for the facilitation of mood-related disorders (Parmigiani et al., 1999; Friedman et al., 2017; Iniguez et al., 2018; Pliota et al., 2020; Qu et al., 2020; Zhang et al., 2021). Early studies of this phenomenon primarily focused on physical stress and frequently neglected emotional stress (Sial et al., 2016; Warren et al., 2020; Qi et al., 2022). To better study the emotional stress, which is characterized by its subjective properties, we developed the three chamber-vicarious social defeat stress (3C-VSDS) model (Qi et al., 2022). In this model, emotionally stressed mice showed anxiety-like behavior but not depression-like behavior; however, behavioral output under the fear-conditioning system (FCS) has not been studied in this model. Aversive learning can profoundly improve animals’ lifetime fitness (Duman and Monteggia, 2006; Kawecki, 2010; Huebner et al., 2018; Namboodiri et al., 2021). Animals learn through association, and associations are reinforced by negative experiences. Failure to learn from negative experiences may have an immediate fatal cost (Yeh et al., 2018; Mason et al., 2021). A previously published study has shown that stress may interfere with associative aversive learning (Grillon, 2002; Stelly et al., 2019; Wu et al., 2021). However, whether emotional stress affects aversive learning is unknown.

In a typical FCS procedure, repeated pairing of a conditional stimulus (CS, e.g., an audible tone) with an unconditional stimulus (US, e.g., foot shock) makes the animal aware of the connection between the CS and the US and allows it to predict the coming foot shock when the CS is delivered, and this elicits a largely automatic conditioned response such as freezing (Duits et al., 2015; Bouton, 2021). The process through which aversive learning occurs commonly consists of two crucial steps, aversive expectation (CS-US pairing elicits the behavioral performance of freezing during the conditioning phase) and aversive outcomes (the CS alone induces freezing during the retrieval phase). Deficits in aversive learning may be related to the maladaptive of aversive expectation (Rescorla, 1988; Maren, 2001).

Decades of research on the neuronal circuits involved in FCS have identified dopamine (DA) neuron systems as the main brain structures associated with FCS in a network of other important brain regions (Likhtik and Johansen, 2019; Pierre et al., 2020; Tang et al., 2020; Tsetsenis et al., 2021). DA is associated with conditioned predictive behavior, particularly given recent evidence that the active avoidance of aversive stimuli requires DA transmission (Wadenberg and Hicks, 1999; Frederic Brischoux et al., 2009; Terao and Mizunami, 2017; Stelly et al., 2019). Moreover, it has been suggested that activation of the mesocorticolimbic pathway, which originates in A10 DA neurons in the ventral tegmental area (VTA), is associated with the contextual fear conditioning (Bariselli et al., 2016). The A13 DA neuron group is located in the zona incerta (ZI), which has comprehensive outputs and inputs to almost the entire neuroaxis (Mitrofanis, 2005; Zhang and van den Pol, 2017; Zhou et al., 2018; Wang X. et al., 2020; Li et al., 2021). Moriya et al. indicated that ZI*^DA^* neurons project to the periaqueductal gray and the central nucleus of the amygdala, two locations that contain key circuits in nociceptive processing, and nociceptive processing is known as an aversive stimulus (Moriya et al., 2020). Brandao et al. also proposed that ZI*^DA^* neurons are the origin of DA neurons that project to caudal structures of the brain in which aversive stimuli are processed (Hasue and Shammah-Lagnado, 2002). These connections and functions of ZI*^DA^* neurons appear to suggest that ZI*^DA^* neurons may be involved in aversive learning.

In this study, to evaluate the effect of emotional stress on aversive learning, *in vivo* calcium fiber photometry and optogenetic technique were used to investigate the role of ZI tyrosine hydroxylase (TH^+^) neurons in wild-type and 3C-VSDS model mice during the FCS procedure. We also sought out the basomedial amygdala (BMA) as the downstream target of ZI TH^+^ neurons and examined the connectivity of the ZI-BMA TH^+^ circuit and its role in FCS. Our findings represent a crucial step in understanding the neural mechanisms underlying aversive expectation and show that the ZI-BMA TH^+^ circuit may rescue aversive learning deficits induced by the 3C-VSDS model.

## Materials and methods

### Animals

All procedures were approved by the Animal Care and Use Committee (Huazhong University of Science and Technology, Wuhan, China) of the university’s animal core facility and performed the following institutional guidelines. We have complied with all relevant ethical regulations for animal testing and research in this study. All mice used in this study were on a C57BL/6J background and given access to food and water *ad libitum*. These mice were group-housed under a 12-h light–dark cycle (lights on at 8:00 a.m.), at consistent humidity (50 ± 5%), and ambient temperature (22 ± 1°C). For behavioral experiments, only male mice (aged 2–2.5 months old at the time of virus injection) were used. All behavioral experiments were conducted during the light cycle.

### Viral vectors

Viruses encoding rAAV2/9-TH-Cre, rAAV2/9-EF1α-DIO-GCaMp6m, rAAV2/9-EF1α-DIO-GFP, rAAV2/9-EF1α-DIO -NpHR3.0-mCherry, rAAV2/9-EF1α-DIO-ChR2-mCherry, rAAV2/9-EF1α-DIO-mCherry, rAAV2/9-hSyn-Cre, rAAV2/9-EF1α-DIO-RVG, rAAV2/9-EF1α-DIO-TVA-EGFP, and RV-ENVA-ΔG-DsRed were purchased from Brain VTA (Brain VTA Co., Ltd., Wuhan, China). The titer of rAAV was in the range of 2–8 × 10^12^ genome copies/ml. Viral vectors were subdivided into aliquots stored at –80°C until use. The following are the abbreviations of viral elements: EF1α, human elongation factor-1 alpha promoter; hSyn, human Synapsin I; NLS, nuclear localization signal; DIO, double-flexed inverted open reading frame.

### Stereotaxic injection

Mice were anesthetized using chloral hydrate (350 mg/kg) and xylazine (10 mg/kg) and then positioned on a stereotactic frame (68030, RWD, China). Body temperature was remained at 37°C using a heating pad. Lidocaine (10 mg/kg) was injected subcutaneously as local anesthesia before incision to the skin. Viruses were injected using a glass micropipette with a tip diameter of 15–20 μm, through a small skull opening (<0.5 mm^2^), with a quintessential stereotaxic injector (Stoelting). After each injection, the syringe was kept in a place for 10 min to allow proper diffusion of the virus. Animals recovered on a heating pad until normal behavior resumed. All experiments involving viral constructs were performed after surgery to allow sufficient expression. The viral infusion coordinate of ZI was shown as follows: anterior–posterior (AP) –2.4 mm, mediolateral (ML) ± 1.6 mm, and dorsal–ventral (DV) –4.2 mm). For the optogenetic inhibition experiment, the mixed viruses containing rAAV2/9-EF1α-DIO-NpHR3.0-mCherry (150 nl, 6.7 × 10^12^ vg/ml) or rAAV2/9-EF1α-DIO-mCherry (150 nl, 2.59 × 10^12^ vg/ml), and AAV2/9-TH-Cre (150 nl, 2.86 × 10^12^ vg/ml) were bilaterally injected into ZI, and optical fibers were bilaterally implanted above ZI or BMA (AP: –1.0 mm, ML: ± 2.55 mm, and DV: –5.2 mm) a week later. For the optogenetic activation experiment, the mixed viruses containing rAAV2/9-EF1α-DIO-hChR2-mCherry (150 nl, 2.85 × 10^12^ vg/ml) or rAAV2/9-EF1α-DIO-mCherry (150 nl, 2.59 × 10^12^ vg/ml), and rAAV2/9-TH-Cre (150 nl, 2.86 × 10^12^ vg/ml) were bilaterally injected into ZI, and optical fibers were bilaterally implanted above BMA a week later. For the retrograde tracing experiment, the mixed viruses containing rAAV2/9-hSyn-Cre (120 nl, 3.31 × 10^12^ vg/ml), rAAV2/9-EF1α-DIO-RVG (120 nl, 2.80 × 10^12^ vg/ml), and rAAV2/9-EF1α-DIO-TVA-EGFP (120 nl, 3.15 × 10^12^ vg/ml) were unilaterally injected into BMA, and RV-ENVA-ΔG-DsRed (80 nl, 4.23 × 10^12^ vg/ml) was ipsilaterally injected into BMA 17 days later. Then, the mice were cardio-perfusion with 0.9% saline followed by phosphate-buffered saline (PBS) containing 4% paraformaldehyde after finishing all behavioral experiments.

### Fiber photometry

For calcium fiber photometry, the mixed viruses containing rAAV2/9-EF1α-DIO-GCaMP6m (120 nl, 4.56 × 10^12^ vg/ml) and rAAV2/9-TH-Cre (120 nl, 2.86 × 10^12^ vg/ml) were unilaterally injected into ZI. After waiting at least 1 week for injection of the virus, the optical fibers (φ1.25 mm, core 200 μm, NA 0.37, length 4.5 mm. Inper Ltd., Hangzhou, China) were unilaterally implanted above ZI. The surface of the skull was sealed with Vetbond (3M). Dental acrylic was used to seal the skull further. Then, 2 weeks after fiber implantation, fluorescence signals were acquired using a fiber photometry system (Inper Ltd., Hangzhou, China) at a frame rate of 20 Hz with an LED power of 10–60% (3–10 mW at the objective, 488 nm). For individual mice, the same acquisition parameters were kept across days. The raw Ca^2+^ fluorescence (F) data were normalized by ΔF/F. F is the averaged fluorescence baseline during a period of 10 s before CS onset (−10–0 s), and ΔF is the signal difference between the fluorescent value at each time point and F. In the habituation and retrieval stages, the ΔF/F were plotted from −10 to 180 s. In the training stage, the ΔF/F was showed from −10 to 40 s for each trial.

### Fear conditioning system

In total, two different contexts were used. Context A (habituation context and fear-conditioning context) contained a clear square chamber (26.1 cm × 26.1 cm) with an electrical grid floor for foot shock delivery, placed in a light-colored sound-attenuating chamber with bright light conditions, and was scented and cleaned with 70% ethanol. Context B (fear retrieval context) consisted of a clear cylindrical chamber (diameter 23 cm) with a smooth floor, placed in a black and white stripes-walled sound-attenuating chamber under dim light conditions. The chamber was scented and cleaned with 1% acetic acid. A stimulus isolator was used for the delivery of a direct current shock. Both chambers contained overhead speakers for the delivery of auditory stimuli. A fear-conditioning device (XR-XZ301, Shanghai Xinruan Information Technology Co., Ltd., Shanghai, China) was used to generate precise pulses to control behavioral protocols. All the signals, including video frame timings, were recorded by the software to synchronize behavioral protocols, behavioral tracking, and video imaging. On Day 1 (habituation phase), the mice were first placed in context A and exposed to a 180-s CS (14-kHz pure tone) following a 3-min baseline period. On Day 2 (fear-conditioning phase), after a 3-min baseline, mice were imaged in context A by pairing five CS (28 s, 14-kHz pure tone) with US (2-s foot shock, 0.7 mA; after the termination of the CS) with an ITI (inter tone interval) of 30 s. Animals remained in context A for 1 min after the last CS-US pairing. On Day 3 (fear retrieval phase), a fear memory was recalled in context B. After a 3-min baseline period, animals were exposed to a 180-s CS.

### Optogenetic manipulation

The light power for optogenetic stimulation (589 nm for NpHR3.0 or 473 nm for ChR2) was adjusted with a power meter (Thorlabs) such that the illumination at the tip of the optic fibers would be at around 10 mW. Constant light (589 nm) stimulation was used to drive the NpHR3.0-mediated inhibition. To sufficiently activate ZI neurons with ChR2, 10-ms pulses were provided at 20 Hz (473 nm). In experiments with optogenetic excitation, the time given to light was only in the late phase (14 s) of the conditioning day. In experiments with optogenetic inhibition, the time given to light was in the late phase (14 s), early phase (14 s), or pre-CS (14 s) of the conditioning day.

### Three chamber-vicarious social defeat stress mouse model

The 3C-VSDS mouse model was performed according to our previously published study (Qi et al., 2022). Briefly, prior to the experiment, CD1 mice were screened on 3 consecutive days and selected according to the following criteria: (i) a latency of attack under 1 min and (ii) attacking for 2 consecutive days. A standard mice cage (55 cm × 40 cm × 20 cm) is trisected by two transparent perforated plexiglas. Experimental male C57BL/6J mice and conspecific male mice were placed on either side of the cage, and aggressive CD1 mice were kept in the center cage. For 10 consecutive days, experimental male C57BL/6J mice watched through transparent perforated plexiglas as conspecific mice were subjected to physical interaction and defeat by aggressive CD1 (resident) for 10 min in center CD1 resident home cage (one defeat stress per day). The conspecific mice were then returned to its cage. Experimental C57BL/6J mice and the conspecific mice were subjected to the continuous psychological stress of sensory interaction with the CD1 aggressors for the duration of the experiment through a transparent perforated plexiglas divider, which allowed aggressive CD1 to interact with experimental male C57BL/6J mice and conspecific mice through visual, auditory, and olfactory for 24 h until the next bout of physical defeat. Experimental male C57BL/6J mice always faced novel conspecific mice and CD1 mice. The control mice were housed and separated by the plexiglas divider barrier and switched each day. They have never seen a physical interaction between conspecific mice and the aggressive CD1. After 10 days of 3C-VSDS, the experimental mice were singly housed and tested 24 h later for open field test and elevated plus maze.

### Open field test

The open field area (50 cm length × 50 cm width × 50 cm height) used in this experiment was made of gray PVC. After the mice were acclimated to the environment room for 2 h, the behavioral test was conducted for 6 min in the open field area. The spontaneous activity of the mice was measured by a video-tracking system (Shanghai Xinruan Information Technology Co., Ltd., Shanghai, China). The time spent in central zone and the total distance travelled in open filed area (total activity) were assessed.

### Elevated plus maze test

The elevated plus maze was composed of two open arms, two closed arms, and a central platform. Each arm had the same length, positioned at 50 cm above the ground. There were two external walls made from white plexiglass (height: 15 cm) for the closed arms. The experimental animals could move freely in the maze. After the mice were acclimated to the room environment for 2 h, the elevated plus maze was conducted for 6 min. The time the animals spent in the open and closed arms was recorded by video-tracking system (Shanghai Xinruan Information Technology Co., Ltd., Shanghai, China). Time spent in the open arms and averaged velocity in elevated plus maze were statistically analyzed.

### Immunofluorescence

Sections (30 μm) of the coronal plane of brain were made using the cryostat microtome and then fixed by using paraformaldehyde (4%). Antigen retrieval assay was carried out by applying sodium dodecyl sulphate (SDS, 1%). After the sections were rinsed by PBS, Triton-X (0.5%) and goat-serum (10%) dissolved in PBS were used to block the non-specific binding between proteins for 1 h. Rabbit polyclonal anti-TH (1:200, 25859-1-AP, Proteintech) dissolved in 5% bovine serum albumin (BSA) was incubated with the sections at 4°C overnight. After rinsing the sections in PBS for three times, fluorochrome-conjugated secondary antibody (1:400, Dylight-594-labeled goat anti-rabbit, Abbikine, CA, United States) was used to incubate with the sections in darkness at room temperature for 1 h. DAPI (1:1000, D9542, Sigma-Aldrich) was then added to stain the nucleus. Coverslips were placed onto the sections after adding the fluorescent mounting medium. The sections were kept in a 4°C environment before observation. Olympus IX-73 microscope and Olympus DP80 photographic equipment (Olympus, Japan) were used to conduct fluorescence imaging for immunofluorescence assessment. Carl Zeiss LSM780 laser scanning confocal microscope (Zeiss Microsystems, Jena, Germany) was used to obtain the images.

### Statistical analysis

The results were expressed as average value ± SEM (*p < 0.05, ^**^p < 0.01, ^***^p < 0.001, ns: no significance). The unpaired two-tailed Student’s *t*-test was conducted to compare the differences between two groups. One-way analysis of variance (ANOVA) with *post hoc* test was conducted for experiments with single factor when the differences among three groups were compared. Two-way repeated measures (RM) ANOVA and multiple comparisons with *post hoc* test were conducted for experiments with double factors. Graphpad Prism and Adobe Illustrator were used for all the analysis and visualization.

## Results

### Zona incerta TH^+^ neurons contribute to aversive expectation in the late stage of fear conditioning

**FIGURE 1:**
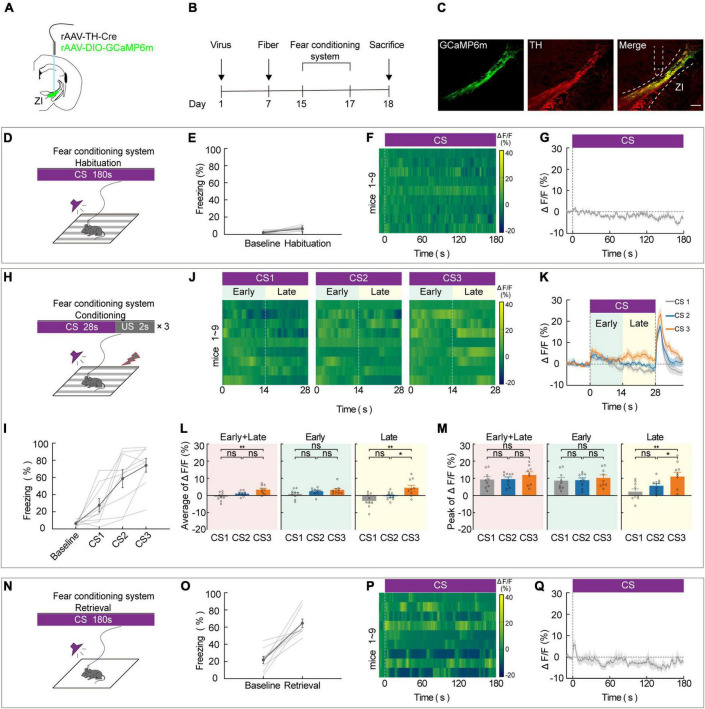
ZI TH^+^ neurons contribute to the aversive expectation in the late stage of CS. **(A)** Schematic representation of the virus injection and optic fiber implantation. **(B)** Experimental protocol timeline. **(C)** Representative image of GCaMP6m expression in the ZI TH-positive cells dominantly; GCaMP6m is shown in green, TH^+^ is shown in red and co-expressed cells are shown in yellow. Scale bar, 500 μm. **(D-G)** Habituation day paradigm. **(D)** Behavioral methodology. **(E)** Freezing behavior during habituation day. (Mean ± SEM. *n* = 9). **(F)** Representative heatmaps of fluorescence aligned to the CS delivery (time point 0) for 9 mice. **(G)** Mean fluorescence aligned to CS delivery (mean ± SEM *n* = 9). **(H-M)** Conditioning phase paradigm. **(H)** Behavioral methodology. **(I)** Freezing behavior during conditioning phase. (Mean ± SEM *n* = 9). **(J)** Representative heatmaps of fluorescence are aligned with CS1, CS2, and CS3 delivery (time point 0) for 9 mice, respectively, arranged from left to right. 0–14 and 14–28 s named early and late phases of CS. **(K)** Mean fluorescence aligned to CS delivery in ZI TH^+^ neurons (mean ± SEM *n* = 9). **(L)** Mean fluorescence to CS3 higher than CS1 delivery for whole session of CS and late phase of CS, but not early phase of CS (early + late: one-way ANOVA, group: *p* = 3 × 10^– 3^; *post hoc* Sidak’s test, CS1 vs. CS2: *p* = 0.1162, CS1 vs. CS3: *p* = 0.0023, CS2 vs. CS3: *p* = 0.0713; early: one-way ANOVA, group: *p* = 0.1492; *post hoc* Sidak’s test, CS1 vs. CS2: *p* = 0.3762, CS1 vs. CS3: *p* = 0.1602, CS2 vs. CS3: *p* = 0.4810; late: one-way ANOVA, group: *p* = 2.6 × 10^– 3^; *post hoc* Sidak’s test, CS1 vs. CS2: *p* = 0.2081, CS1 vs. CS3: *p* = 0.0023, CS2 vs. CS3: *p* = 0.0340; mean ± SEM n = 9). **(M)** Peak fluorescence to CS3 higher than CS1 delivery for late phase of CS, but not early phase of CS (early + late: one-way ANOVA, group: *p* = 0.4555; *post hoc* Sidak’s test, CS1 vs. CS2: *p* = 0.9134, CS1 vs. CS3: *p* = 0.5939, CS2 vs. CS3: *p* = 0.5939; early: one-way ANOVA, group: *p* = 0.7558; *post hoc* Sidak’s test, CS1 vs. CS2: *p* = 0.9351, CS1 vs. CS3: *p* = 0.8730, CS2 vs. CS3: *p* = 0.8730; late: one-way ANOVA, group: *p* = 2.5 × 10^– 3^; *post hoc* Sidak’s test, CS1 vs. CS2: *p* = 0.1740, CS1 vs. CS3: *p* = 0.0021, CS2 vs. CS3: *p* = 0.0393; mean ± SEM n = 9). **(N–Q)** Retrieval day paradigm. **(N)** Behavioral methodology. **(O)** Freezing behavior during the retrieval phase. (Mean ± SEM *n* = 9). **(P)** Representative heatmaps of fluorescence aligned to the CS delivery (time point 0) for 9 mice. **(Q)** Mean fluorescence aligned to CS delivery (mean ± SEM *n* = 9). ns not significant, *P ≤ 0.05, **P ≤ 0.01.

To assess the effect of ZI*^DA^* neurons on aversive associative learning, we used *in vivo* calcium fiber photometry to measure the activity of ZI TH^+^ neurons during each phase of FCS. During this test, repeated pairing of a CS with a US causes the animal to become aware of the connection between them, and this elicits a largely automatic conditional response such as freezing. The frequency of freezing was used to assess the aversive learning ability of the mice. To selectively record the calcium activity of ZI TH^+^ neurons during this task, we unilaterally injected a virus that contains a recombinant adeno-associated virus (rAAV) that expresses Cre recombinase in TH-positive (TH-Cre) neurons and a Cre-dependent calcium sensor (rAAV2/9-EF1α-DIO-GCaMP6 m). A week later, optical fibers were unilaterally implanted in the ZI (Figures 1A,B). After 2 weeks of virus expression, the calcium activity of ZI-infected cells was recorded during the 3-day FCS paradigm, which included habituation, conditioning, and retrieval phases. We used immunofluorescence labeling of TH antibody to verify that the GCaMP6m was specifically expressed in ZI TH^+^ neurons (Figure 1C and Supplementary Figure 1). Correct viral targeting and fiber positioning were verified after sacrifice (Supplementary Figure 2). When CS was administered during the habituation phase (Day 1), neither the freezing level (Figures 1D,E) nor the calcium fluorescence (Figures 1F,G) changed significantly compared to baseline, which indicated that ZI TH^+^ neurons did not respond significantly to the CS tone. During fear conditioning (Day 2), three pairings of an auditory CS with a US (a 0.7 mA footshock) were administered (Figure 1H). CS-elicited freezing increased during the conditioning phase (Figure 1I). In addition, *in vivo* calcium optic fiber recording—which simultaneously measured the activity of ZI TH^+^ neurons—showed that the average values of calcium fluorescence (ΔF/F) in ZI TH^+^ neurons increased gradually during the CS, especially during the late stage of CS administered among the three CS-US pairs (Figures 1J,K). Furthermore, the mean and peak values of ΔF/F of the CS-US pairs were significantly higher in the 3rd CS (CS3) than in the 1st CS (CS1) during the whole session (early + late) and during the late stage but not during the early stage of CS (Figures 1L,M). Meanwhile, mice with ZI TH^+^ neurons expressing GFP showed no changes during FCS to exclude movement impact (Supplementary Figure 3). On retrieval day (Day 3), an increased level of freezing, a readout of aversive learning, was significantly induced by the CS during the retrieval phase (Figures 1N,O). This indicated that the mice had successfully learned to associate the CS and with the US. The calcium signals of ZI TH^+^ neurons at the initial stage when the animals were given the retrieval CS also increased markedly (Figures 1P,Q). These results indicate that the activity of ZI TH^+^ neurons increased significantly during the conditioning phase of the FCS, especially during the late stage of the CS, suggesting that ZI TH^+^ neurons play a crucial role in aversive expectation.

### Optogenetic inhibition of zona incerta TH^+^ neurons disrupts conditioned fear learning in mice

**FIGURE 2:**
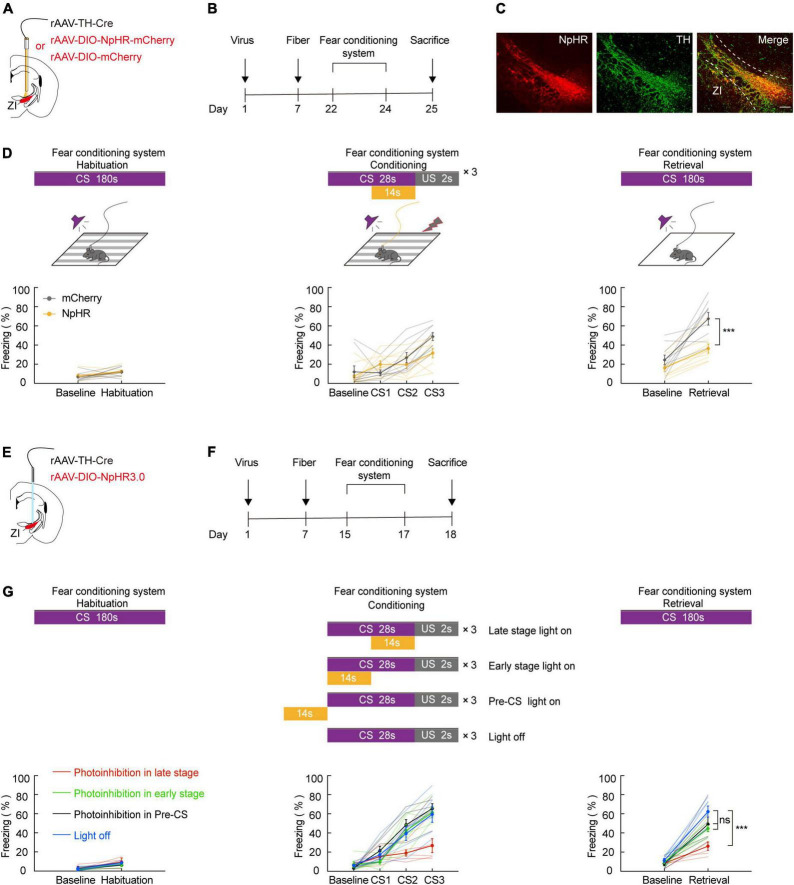
Photogenetic inhibition of ZI TH^+^ neurons in the late stage of CS disrupted conditioned fear learning in mice. **(A)** Schematic representation of the virus injection and optic fiber implantation. **(B)** Experimental protocol timeline. **(C)** Representative image of NpHR3.0-mCherry expression in the ZI TH-positive cells dominantly. NpHR3.0 is shown in red, TH + is shown in green, and co-expressed cells are shown in yellow. Scale bar, 500 μm. **(D)** Behavioral paradigm of fear-conditioning system and its corresponding freezing behavior (habituation phase, two-way RM ANOVA, group: *p* = 0.5041, time: *p* = 0.0033, interaction: *p* = 0.8917; *post hoc* Sidak’s test, Baseline: mCherry vs. NpHR3.0: *p* = 0.8144; Habituation: mCherry vs. NpHR3.0: *p* = 0.9130. Conditioning phase, two-way RM ANOVA, group: *p* = 0.2849, time: *p* < 0.0001, interaction: *p* = 0.0146; *post hoc* Sidak’s test, baseline: mCherry vs. NpHR3.0: *p* = 0.9645; CS1: mCherry vs. NpHR3.0: *p* = 0.3209. CS2: mCherry vs. NpHR3.0: *p* = 0.8012; CS3: mCherry vs. NpHR3.0: *p* = 0.0693. Retrieval phase, two-way RM ANOVA, group: *p* = 0.0012, time: *p* < 0.0001, interaction: *p* = 0.0467; *post hoc* Sidak’s test, baseline: mCherry vs. NpHR3.0: *p* = 0.4568; retrieval: mCherry vs. NpHR3.0: *p* = 0.0003; mean ± SEM n = 9). **(E)** Schematic representation of the virus injection and optic fiber implantation. **(F)** Experimental protocol timeline. **(G)** Behavioral paradigm of fear-conditioning system and its corresponding freezing behavior (retrieval phase, two-way RM ANOVA, group: *p* = 0.0008, time: *p* < 0.0001, interaction: *p* = 0.0103; *post hoc* Sidak’s test, retrieval: late stage light on vs. early stage light on: *p* = 0.0020; late stage light on vs. pre-CS light on: *p* = 0.0240; late stage light on vs. light off: *p* < 0.0001; early stage light on vs. pre-CS light on: *p* = 0.9487; early stage light on vs. light off: *p* = 0.2049; pre-CS light on vs. light off: *p* = 0.0260. Mean ± SEM *n* = 6). ns not significant, ***P ≤ 0.001.

Our fiber photometry data indicate that during the course of conditioning, there is an expected increase in the late stage of CS. Thus, we hypothesized that *in vivo* inhibition of ZI TH^+^ neurons would be sufficient to reverse CS-US pairings. To investigate this, we utilized optogenetics in combination with FCS administration. To selectively inhibit ZI TH^+^ neurons, we bilaterally injected a mixed virus that contained rAAV2/9-TH-Cre and encoded a Cre-dependent inhibitory halorhodopsin (rAAV2/9-DIO-NpHR3.0-mCherry) or a control virus (rAAV-DIO-mCherry) into the ZI and then bilaterally implanted optical fibers above the ZI (Figures 2A,B). First, we demonstrated by immunofluorescence that both control and NpHR3.0 viral expression were highly restricted to TH^+^ neurons in the ZI (Figure 2C and Supplementary Figure 4). We found that inhibition of NpHR3.0-expressing TH^+^ neurons by laser stimulation during each late stage of the conditioning phase was sufficient to decrease the CS-induced freezing level during the retrieval phase compared to the level observed in the mCherry group (Figure 2D). Notably, the NpHR3.0 group also showed a reduced freezing level in CS3 trial during the conditioning phase. Next, we verified whether inhibition of the ZI TH^+^ neurons only in the late stage of CS could affect aversive learning. We bilaterally injected a mixed virus that contained rAAV2/9-TH-Cre and rAAV2/9-DIO-NpHR3.0-mCherry into the ZI and then bilaterally implanted optical fibers above the ZI (Figures 2E,F). After 2 weeks of virus expression, four groups of mice were subjected to the FCS system, and the time given to light was in the late phase (14 s), early phase (14 s), pre-CS (14 s), or given no light of the conditioning day (Figure 2G). The results showed that only inhibit the ZI TH^+^ neurons in the late stage of CS could decrease the CS-induced freezing during the retrieval phase compared to no light group. Together, these data suggested that the optogenetic deactivation of ZI TH^+^ neurons in the late stage of CS disturbs the conditioning of cued-based fear memory, indirectly proving that ZI TH^+^ neurons play an indispensable role in aversive associative learning.

### Zona incerta TH^+^ neurons project to the basomedial amygdala, and optogenetic inhibition of the zona incerta-basomedial amygdala TH^+^ circuit blocks aversive learning

**FIGURE 3:**
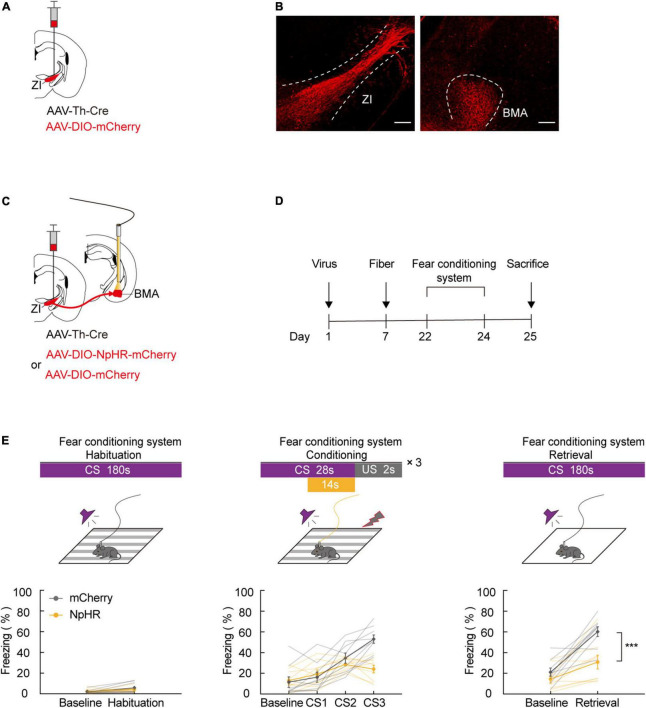
ZI TH^+^ neurons projected to BMA, and photogenetic inhibition of ZI-BMA TH^+^ circuit blocked aversive learning. **(A)** Schematic representation of the virus injection. **(B)** Representative image of mCherry in the ZI injection site and mCherry^+^ terminals in the BMA. Scale bar, 500 μm. **(C)** Schematic representation of the virus injection and optic fiber implantation. **(D)** Experimental protocol timeline. **(E)** Behavioral paradigm of fear-conditioning system and its corresponding freezing behavior (habituation phase, two-way RM ANOVA, group: *p* = 0.6091, time: *p* = 0.0127, interaction: *p* = 0.2868; *post hoc* Sidak’s test, baseline: mCherry vs. NpHR3.0: *p* = 0.9341; Habituation: mCherry vs. NpHR3.0: *p* = 0.4705. Conditioning phase, two-way RM ANOVA, group: *p* = 0.1260, time: *p* < 0.0001, interaction: *p* < 0.0001; *post hoc* Sidak’s test, baseline: mCherry vs. NpHR3.0: *p* = 0.9987; CS1: mCherry vs. NpHR3.0: *p* = 0.9865. CS2: mCherry vs. NpHR3.0: *p* = 0.7015; CS3: mCherry vs. NpHR3.0: *p* = 0.0003. Retrieval phase, two-way RM ANOVA, group: *p* = 0.0035, time: *p* < 0.0001, interaction: *p* = 0.0238; *post hoc* Sidak’s test, baseline: mCherry vs. NpHR3.0: *p* = 0.5714; retrieval: mCherry vs. NpHR3.0: *p* = 0.0004. Mean ± SEM *n* = 9). ns not significant, ***P ≤ 0.001.

Our previous results indicate that ZI TH^+^ neurons are involved in aversive expectation. The amygdala is known as a predominant component of the neural circuit that regulates fear-related behavior. To investigate whether ZI TH^+^ neurons project to the amygdala, we unilaterally injected a mixed virus containing rAAV2/9-TH-Cre and rAAV2/9-DIO-mCherry into the ZI (Figure 3A). After 3 weeks of virus expression, we determined whether the amygdala region contained the axon terminals of ZI TH^+^ neurons. The injection site of virus expression in the ZI and the mCherry-positive terminals in the BMA are shown in Figure 3B and Supplementary Figure 5. Then, we adopted retrograde tracing strategy to reconfirm the ZI-BMA circuit. We unilaterally injected a mixed virus containing rAAV2/9-hSyn-Cre, rAAV2/9-EF1α-DIO-RVG, and rAAV2/9-EF1α-DIO-TVA-EGFP into the BMA, RV-ENVA-ΔG-DsRed was ipsilaterally injected into BMA 17 days later, and DsRed-labeled neurons were found in the ZI traced from the BMA neurons (Supplementary Figure 6).

Next, we tested whether inhibiting the ZI TH^+^ neuronal circuit projecting to the BMA was sufficient to cause a deficiency in FCS. We bilaterally injected a mixed virus containing rAAV2/9-TH-Cre and rAAV2/9-DIO-NpHR3.0-mCherry or the control virus, rAAV2/9-DIO-mCherry, into the ZI and then bilaterally implanted optical fibers above the BMA in such a way that the fibers could be used to specifically inhibit activity at the axon terminals of BMA-projecting ZI TH^+^ neurons (Figures 3C,D). During FCS, we highly selectively inhibited the activity of NpHR3.0-expressing axon projections in the ZI-BMA circuit by continuous yellow light illumination. By utilizing this approach, we found that inhibition of the ZI-BMA TH^+^ circuit with a yellow laser (589 nm) during the late stages of three CS-US pairings robustly reduced the freezing level during CS3 in the conditioning phase and during CS administration in the retrieval phase compared to the control group (Figure 3E). These findings show that ZI TH^+^ neurons project to the BMA and that this TH^+^ circuit is required for the conditioned fear response and aversive associative learning.

### Three chamber-vicarious social defeat stress model mice show suppresses of zona incerta TH^+^ neuron activity during the late stage of conditional stimulus

**FIGURE 4:**
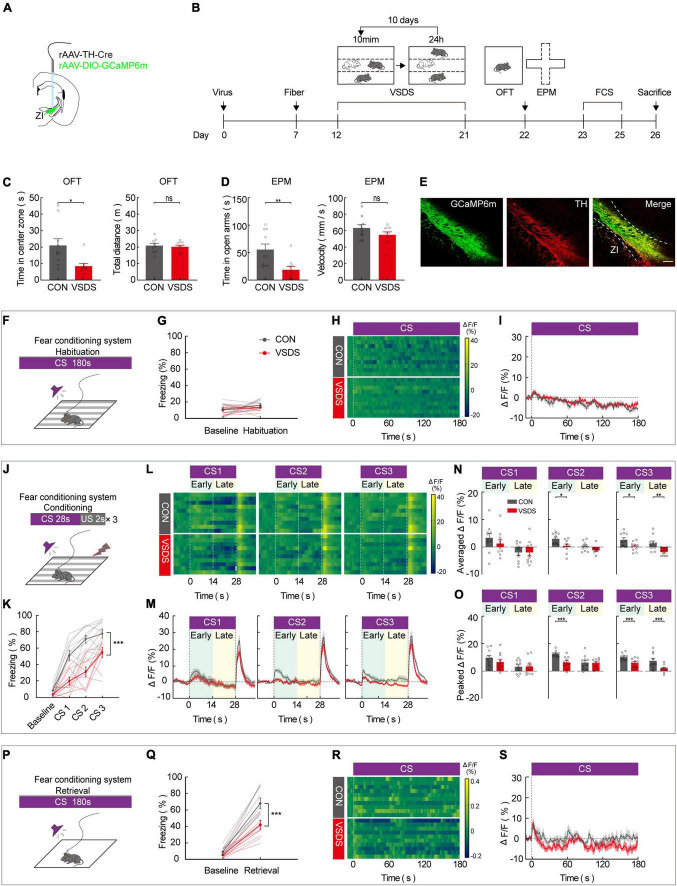
3C-VSDS suppresses ZI TH^+^ neuron activity during the late stage of CS. **(A)** Schematic representation of the virus injection and optic fiber implantation. **(B)** Experimental protocol timeline. **(C)** Bar graphs represent time in center zone and total distance, respectively, by open field test in control with VSDS mice (unpaired t test, time in the center zone: *p* = 0.0108, total distance: *p* = 0.6842. Mean ± SEM *n* = 10). **(D)** Bar graphs represent time in open arm and average velocity, respectively, by EPM test in control with VSDS mice (unpaired *t*-test, time in open arm: *p* = 0.0055, average velocity: *p* = 0.2475. Mean ± SEM *n* = 10). **(E)** Representative image of GCaMP6m expression in the ZI TH-positive cells dominantly; GCaMP6m is shown in green, TH^+^ is shown in red and co-expressed cells are shown in yellow. Scale bar, 500 μm. **(F-I)** Habituation day paradigm. **(F)** Behavioral methodology. **(G)** Freezing behavior of control and VSDS mice on habituation day. (Two-way RM ANOVA, group: *p* = 0.3360, time: *p* = 0.1371, interaction: *p* = 0.7738; *post hoc* Sidak’s test, baseline: Con vs. VSDS: *p* = 0.9426; habituation: Con vs. VSDS: *p* = 0.6790. Mean ± SEM n = 10). **(H)** Representative heatmaps of fluorescence of control and VSDS mice aligned with the CS delivery (time point 0) for 10 mice. **(I)** Mean fluorescence of control and VSDS mice aligned with the CS delivery (mean ± SEM n = 10). **(J-O)** Conditioning phase paradigm. **(J)** Behavioral methodology. **(K)** Freezing behavior of control and VSDS mice on conditioning phase. (Two-way RM ANOVA, group: *p* < 0.0001, time: *p* < 0.0001, interaction: *p* = 0.0041; *post hoc* Sidak’s test, baseline: Con vs. VSDS: *p* = 0.1876; CS1: Con vs. VSDS: *p* = 0.0051. CS2: Con vs. VSDS: *p* = 0.0003. CS3: Con vs. VSDS: *p* = 0.0191. Mean ± SEM *n* = 10). **(L)** Representative heatmaps of fluorescence of control and VSDS mice are aligned with CS1, CS2, and CS3 delivery (time point 0) for 10 mice, respectively, arranged from left to right. 0–14 and 14–28 s named early and late phases of CS. **(M)** Mean fluorescence of control and VSDS mice aligned to CS delivery in ZI TH^+^ neurons (mean ± SEM n = 10). **(N)** Bar graphs represent average fluorescence of control and VSDS mice at the early and late phase of CS1, CS2, and CS3, respectively, arranged from left to right (unpaired *t*-test, CS1 early, Con vs. VSDS: *p* = 0.3470, CS1 late, Con vs. VSDS: *p* = 0.9838; CS2 early, Con vs. VSDS: *p* = 0.0124, CS2 late, Con vs. VSDS: *p* = 0.0860; CS3 early, Con vs. VSDS: *p* = 0.0189, CS3 late, Con vs. VSDS: *p* = 0.0044; mean ± SEM *n* = 10). **(O)** Bar graphs represent peak fluorescence of control and VSDS mice at the early and late phase of CS1, CS2, and CS3, respectively, arranged from left to right (unpaired *t*-test, CS1 early, Con vs. VSDS: *p* = 0.2938, CS1 late, Con vs. VSDS: *p* = 0.9493; CS2 early, Con vs. VSDS: *p* = 0.0007, CS2 late, Con vs. VSDS: *p* = 0.8252; CS3 early, Con vs. VSDS: *p* = 0.0035, CS3 late, Con vs. VSDS: *p* = 0.0085; mean ± SEM *n* = 10). **(P-S)** Retrieval phase paradigm. **(P)** Behavioral methodology. **(Q)** Freezing behavior of control and VSDS mice on retrieval phase. (Two-way RM ANOVA, group: *p* = 0.0064, time: *p* < 0.0001, interaction: *p* = 0.0120; *post hoc* Sidak’s test, baseline: Con vs. VSDS: *p* = 0.8053; retrieval: Con vs. VSDS: *p* = 0.0004. Mean ± SEM *n* = 10). **(R)** Representative heatmaps of fluorescence of control and VSDS mice aligned with the CS delivery (time point 0). **(S)** Mean fluorescence of control and VSDS mice aligned with the CS delivery (mean ± SEM *n* = 10). ns not significant, *P ≤ 0.05, **P ≤ 0.01, ***P ≤ 0.001.

Previous work has demonstrated that the effect of emotional stress on anxiety and depression behavior can be studied using the 3C-VSDS model, but the model has not been used to study the effect of emotional stress on aversive learning. We further investigated the behavioral performance of both control and 3C-VSDS model mice during FCS and simultaneously recorded calcium activity in ZI TH^+^ neurons. We unilaterally injected a mixed virus that contained rAAV2/9-TH-Cre and rAAV2/9-DIO-GCaMP6 m into ZI and then unilaterally implanted optical fibers above the ZI (Figures 4A,B). After 12 days of virus expression, the model mice were exposed to 10 consecutive days of 3C-VSDS. After 10 days of 3C-VSDS treatment, the model mice were tested using the open field test (OFT). Relative to the control animals, the 3C-VSDS mice showed a shorter duration of center exploration without affecting the total distance (Figure 4C). We also subjected mice to the elevated plus maze test (EPM); the results of that test showed that the 3C-VSDS mice spent less time in open-arm exploration without affecting the average velocity (Figure 4D), consistent with our OFT results. Thus, the 3C-VSDS model reliably induces an array of anxiety-like phenotypes in mice.

Immunofluorescence staining indicated that GCaMP6 m was highly restricted to TH-positive neurons in the ZI (Figure 4E). After 3 weeks of virus expression, the calcium activity of ZI-infected neurons was recorded during the 3-day FCS paradigm in both control and 3C-VSDS mice. During the habituation phase, there was no significant difference between control mice and 3C-VSDS mice in freezing level (Figures 4F,G) or calcium fluorescence when the CS was administered (Figures 4H,I). Next, we analyzed the animals’ behavioral performance and calcium fluorescence in the fear-conditioning phase, which consisted of three pairings of an auditory CS with a footshock US. Compared to control mice, 3C-VSDS mice displayed a lower freezing level (Figures 4J,K). *In vivo* fiber photometry was performed simultaneously to detect the activity of ZI TH^+^ neurons during the behavioral test. We then analyzed the average and peak calcium fluorescence (ΔF/F) of ZI TH^+^ neurons in the control mice and the 3C-VSDS model mice. The 3C-VSDS group showed significantly lower peak values than the control group in response to the CS during the conditioning phase, including the early stage of the 2nd CS (CS2) and both the early and late stages of the 3rd CS (CS3) (Figures 4L–O). Interestingly, the ΔF/F of calcium fluorescence in the early stages of CS2 and CS3 also showed significant differences between control and 3C-VSDS model mice, indicating that 3C-VSDS model mice perhaps had reduced sensitivity to CS cues. During the retrieval phase, the freezing level, the outcome of aversive associative learning, was also significantly decreased in the 3C-VSDS group compared with the control group (Figures 4P,Q). The calcium activity of ZI TH^+^ neurons showed no significant difference in response to CS recall during the retrieval phase between 3C-VSDS mice and control mice (Figures 4R,S). These results suggest that ZI TH^+^ neurons are involved in the conditioning phase of FCS. Additionally, the decreased activity of ZI TH^+^ neurons during the late stage of CS may be related to a phenotype with aversive associative learning deficits in 3C-VSDS mice.

### Optogenetic activation of the zona incerta-basomedial amygdala TH^+^ circuit markedly relieves three chamber-vicarious social defeat stress-induced aversive learning disabilities

**FIGURE 5:**
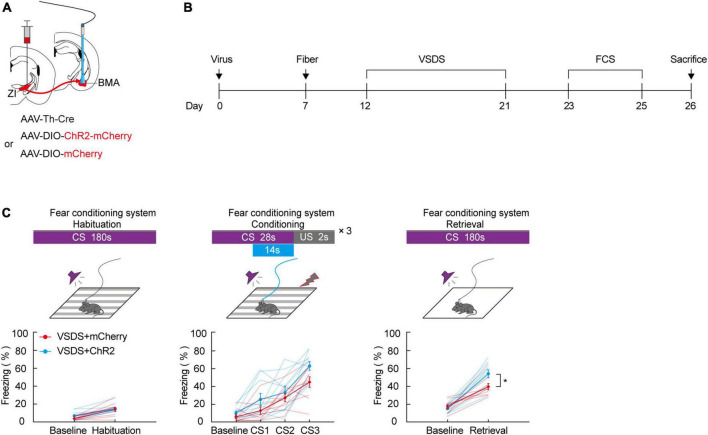
Photogenetic activation of ZI-BMA TH^+^ circuit remarkedly relieve 3C-VSDS-induced aversive associative learning disabilities. **(A)** Schematic representation of the virus injection and optic fiber implantation. **(B)** Experimental protocol timeline. **(C)** Behavioral paradigm of fear-conditioning system and its corresponding freezing behavior (habituation phase, two-way RM ANOVA, group: *p* = 0.3253, time: *p* < 0.0001, interaction: *p* = 0.3456; *post hoc* Sidak’s test, baseline: VSDS + mCherry vs. VSDS + ChR2: *p* = 0.3238; habituation: VSDS + mCherry vs. VSDS + ChR2: *p* = 0.9361. Conditioning phase, two-way RM ANOVA, group: *p* = 0.0572, time: *p* < 0.0001, interaction: *p* = 0.3033; *post hoc* Sidak’s test, baseline: VSDS + mCherry vs. VSDS + ChR2: *p* = 0.8166; CS1: VSDS + mCherry vs. VSDS + ChR2: *p* = 0.5307. CS2: VSDS + mCherry vs. VSDS + ChR2: *p* > 0.9999; CS3: VSDS + mCherry vs. VSDS + ChR2: *p* = 0.1143. Retrieval phase, two-way RM ANOVA, group: *p* = 0.0306, time: *p* < 0.0001, interaction: *p* = 0.0223; *post hoc* Sidak’s test, baseline: VSDS + mCherry vs. VSDS + ChR2: *p* = 0.9934; retrieval: VSDS + mCherry vs. VSDS + ChR2: *p* = 0.0032. Mean ± SEM *n* = 10). *P ≤ 0.05.

Our previous results in this study demonstrate that aversive associative learning impairments caused by 3C-VSDS may be linked to the activity of ZI TH^+^ neurons. Therefore, we determined whether photoactivation of the TH^+^ circuit from the ZI to the BMA in 3C-VSDS mice could reverse the deficits in aversive associative learning. To do this, we bilaterally injected a mixed virus containing rAAV2/9-TH-Cre and the excitatory opsin channelrhodopsin-2 (ChR2) rAAV2/9-DIO-ChR2-mCherry or the control virus rAAV2/9-DIO-mCherry into the ZI and then bilaterally implanted above the BMA optical fibers that could be used to specifically activate the axon terminals of BMA-projecting ZI TH^+^ neurons (Figures 5A,B). The results showed that optogenetic activation of the TH^+^ ZI-BMA circuit during the late stage of the conditioning phase robustly increased the freezing level in the 3C-VSDS + ChR2 group compared to the 3C-VSDS + mCherry group (Figure 5C). The freezing level was enhanced both during CS3 of the conditioning phase and during CS-elicited fear recall during the retrieval phase. Taken together, these findings further indicated that the TH^+^ circuit from the ZI to the BMA is required for aversive associative learning in 3C-VSDS –induced aversive learning deficits.

## Discussion

Here, we reported that the activity of ZI TH^+^ neurons is significantly increased by delivery of repeated CS-US pairings during the entire duration or the late stage of the CS but that this does not occur when the pairings are delivered during its early stages. The increased activity of ZI TH^+^ neurons is at least in part related to aversive expectation during the process of aversive learning in which the animal learns that an aversive shock stimulus will follow the CS. Inhibition of the activity of ZI TH^+^ neurons at the late stage of the conditioning phase by photogenetic techniques resulted in a lower level of freezing, implying an inappropriate aversive expectation compared to the control. We then identified the BMA as a downstream target of ZI TH^+^ neurons and found that photoinhibition of the axon terminals in the ZI-BMA TH^+^ circuit during the late-stage also affected the aversive expectation of association learning in FCS. Our 3C-VSDS model mice showed a significant reduction in freezing level compared with the control group, and the model mice exhibited an obvious deficit in aversive learning. Correspondingly, 3C-VSDS mice displayed decreased activity in ZI TH^+^ neurons during the conditioning phase of FCS. Based on these and the previous results, we hypothesized that ZI TH^+^ neurons and the ZI-BMA TH^+^ circuit might be involved in aversive learning deficits in 3C-VSDS model mice. Optogenetic activation of the ZI-BMA TH^+^ circuit indeed ameliorates 3C-VSDS-induced aversive learning deficits, as indicated by the low freezing level. Our results provide insight into the role of the ZI TH^+^ neurons and their output pathway, the ZI-BMA TH^+^ circuit, in aversive learning, especially in aversive expectation during FCS.

Our results suggest that activity of ZI TH^+^ neurons is essential for the development of aversive learning, especially for the development of aversive expectation. A recent study by Li et al. showed increased activity in lateral habenula (LHb) neurons when individuals receive stimuli that predict aversive conditions. LHb neurons project and modulate DA-rich regions in the VTA (Li et al., 2011). Cai et al. revealed a neural loop connection between ZI and VTA DA neurons (Cai et al., 2022). ZI TH^+^ neurons can regulate aversive expectations, and this may be related to upstream projections. The results we obtained using calcium fluorescence also showed that the activity of ZI TH^+^ neurons increased significantly during aversive expectation learning, specifically during the late stage of CS delivery. Optogenetic inhibition of the activity of ZI TH^+^ neurons and the ZI-BMA TH^+^ circuit at the late stage of CS has been used to delineate their role in mediating aversive expectation learning. Taken together, these results show that increased activity of ZI TH^+^ neurons and of the ZI-BMA TH^+^ circuit is at least in part related to aversive expectation during the process of associative learning in which animals learn that an aversive shock stimulus will follow a CS.

We found that mice that were subjected to chronic emotional stress in the 3C-VSDS model showed a deficit in aversive learning. The activity of ZI TH^+^ neurons was significantly lower in 3C-VSDS model mice during conditioning stage than in control mice, especially during the late stage of CS3. We suggest that ZI TH^+^ neurons participate in aversive learning in addition to their involvement in aversive learning deficits in 3C-VSDS model mice. The fact that chronic stress can induce severe cognitive impairment has been widely demonstrated in humans and animals (Roozendaal et al., 2009; Marin et al., 2011; McEwen et al., 2015; Jia et al., 2021). Our 3C-VSDS model allows us to study the effects of chronic(10-d) emotional stress independently of physical stress. During the 10-day continuous procedure, the model mice observed aggressive CD1 mice violently attacking their conspecifics; through witnessing the intense emotional stress that was occurring in the animals next door, these mice experienced emotional stress in which they shared the emotional states of their conspecifics (Qi et al., 2022). Jonsdottir et al. (2013) reported that patients with stress-related exhaustion showed cognitive impairment relative to healthy controls and that these patients performed worse on tasks involving attention, working memory, contextual memory, and learning. Our results indicate that model mice have deficits in aversive learning following exposure to prolonged emotional stress. Optogenetic excitation of the ZI-BMA TH^+^ circuit at the late stage of CS successfully reversed aversive learning deficits in 3C-VSDS model mice, indicating that the learning deficits observed in 3C-VSDS model mice may be caused by abnormal levels of aversive expectations. Interestingly, 3C-VSDS model mice also showed an anxious phenotype; whether the aversive learning deficits observed in these animals are caused by anxiety or by stress-related exhaustion needs to be further explored in the future studies.

Jia et al. (2021) recently showed that chronic stress induces dramatic deficits in learning and memory in *Drosophila* models and found that the dopaminergic system in *Drosophila* plays an important role in regulating chronic stress-induced learning deficits. This suggests that DA neurons may play a crucial role in learning deficits. Thus, it is reasonable to investigate the role of ZI*^DA^* neurons in the regulation of aversive learning deficits in emotion-stressed mice. It is known that midbrain DA neurons are strongly activated when the differences occur between predicted and actual outcomes, a situation that is referred to as prediction errors, and these neuronal populations are thought to be responsible for associative learning (Steinberg et al., 2013; Chang et al., 2016). Increased activity of ZI TH^+^ neurons during aversive learning may partially be a result of prediction errors regarding unexpected US, and such prediction errors are officially the basis of association learning. Chou et al. discovered that the activation of ZI GABAergic neurons or of their projection to the PAG can reduce conditioned freezing behaviors. Whether DA and GABA neurons in the ZI have local connections is unknown. If they do, ZI TH^+^ neurons may be regulated by local GABAergic neurons; further research on this point is needed. Wang et al. reported that parvalbumin (PV)-positive neuronal projections from the ZI to the Po (ZI-Po) are critical for promoting nocifensive behaviors (Wang H. et al., 2020). We cannot rule out the possibility that ZI TH^+^ neurons have local connections with GABA neurons that modulate freezing behavior through nocifensive adjustment (Chou et al., 2018; Venkataraman et al., 2021).

In this study, we used optogenetic techniques to regulate the activity of ZI TH^+^ neurons and their projection to the BMA, eNpHR3.0 is the most suitable existing tool for the study of synaptic modulation, although its use should be carefully controlled to account for changes in chloride reversal potential and strong light-off rebound responses. As Mahn et al. show, rebound excitation is prevented by reducing the intensity of the light over a period of tens of milliseconds (Mahn et al., 2016; Wiegert and Oertner, 2016). *In vivo* calcium fiber photometry was also used to record the activity of ZI TH^+^ neurons; in this process, the systematic error of photobleaching during the fluorescence signal capture should be considered (Wu et al., 2012). Another consideration is that we used only male mice in our experiments; thus, further study is needed to determine whether the same results can be obtained in female mice. Finally, which type of neurotransmitter (noradrenaline or dopamine) and which associated receptors are the effectors of ZI TH^+^ neurons remain to be established.

In this work, we investigated the function of ZI TH^+^ neurons and the ZI-BMA TH^+^ projection in aversive expectation in associative learning as well as in 3C-VSDS-induced aversive expectation deficits. In future experiments, we would like to further study whether GABAergic populations of ZI neurons affect aversive expectation. In conclusion, we propose that changes in the ZI-BMA TH^+^ pathways are a crucial factor in improving aversive associative learning deficits. Our findings provide important mechanistic insight into the neural circuitry underlying the aversive expectation of associative learning at baseline and under conditions of emotional stress.

## Data availability statement

The original contributions presented in this study are included in the article/Supplementary material, further inquiries can be directed to the corresponding author.

## Ethics statement

The animal study was reviewed and approved by Animal Care and Use Committee, Huazhong University of Science and Technology, Wuhan, China.

## Author contributions

BT, JM, and PZ conceived the idea and design for the study. LZ and GQ contributed to study design. LZ and PZ performed the experiments, analyzed the data, and wrote the manuscript. HC, TL, ML, CC, JL, KR, and JY contributed to the final version of the manuscript. All authors contributed to the article and approved the submitted version.
