# Supplementary material for: A zona incerta-basomedial amygdala circuit modulates aversive expectation in emotional stress-induced aversive learning deficits
Source: Front Cell Neurosci. 2022 Aug 26;16:910699. doi: 10.3389/fncel.2022.910699 (PMC9459227; doi:10.3389/fncel.2022.910699)
Supplement: Supplementary file 1 [file Data_Sheet_1.pdf]

A

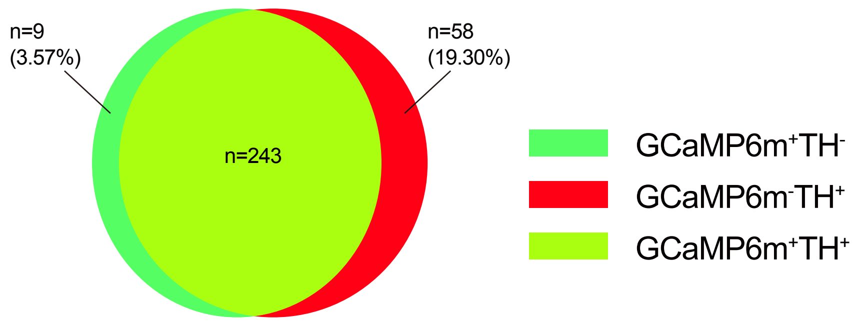

Supplementary Figure 1 | The quantitative analysis of Venn diagram shows the co-expression level of GCaMP6m with TH in ZI.

(A) The quantitative analysis of Venn diagram shows the co-expression level of GCaMP6m with TH in ZI (3 sections per mouse from 3 mice).

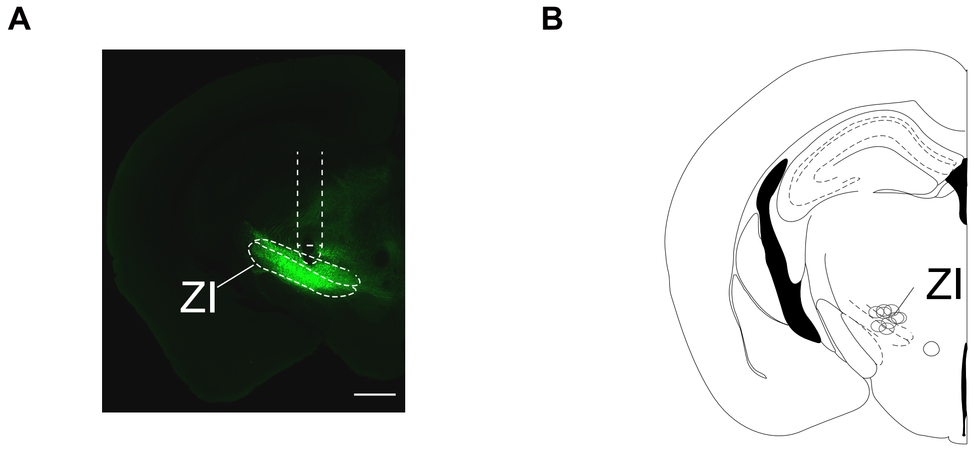

Supplementary Figure 2 | Probe placement map into ZI.

(A) Histological example showing an optic fiber placement in the ZI. Green: GCaMP6m. Scale bar, 500μm.

(B) Schematic coronal sections showing the location of the recording sites in the ZI.

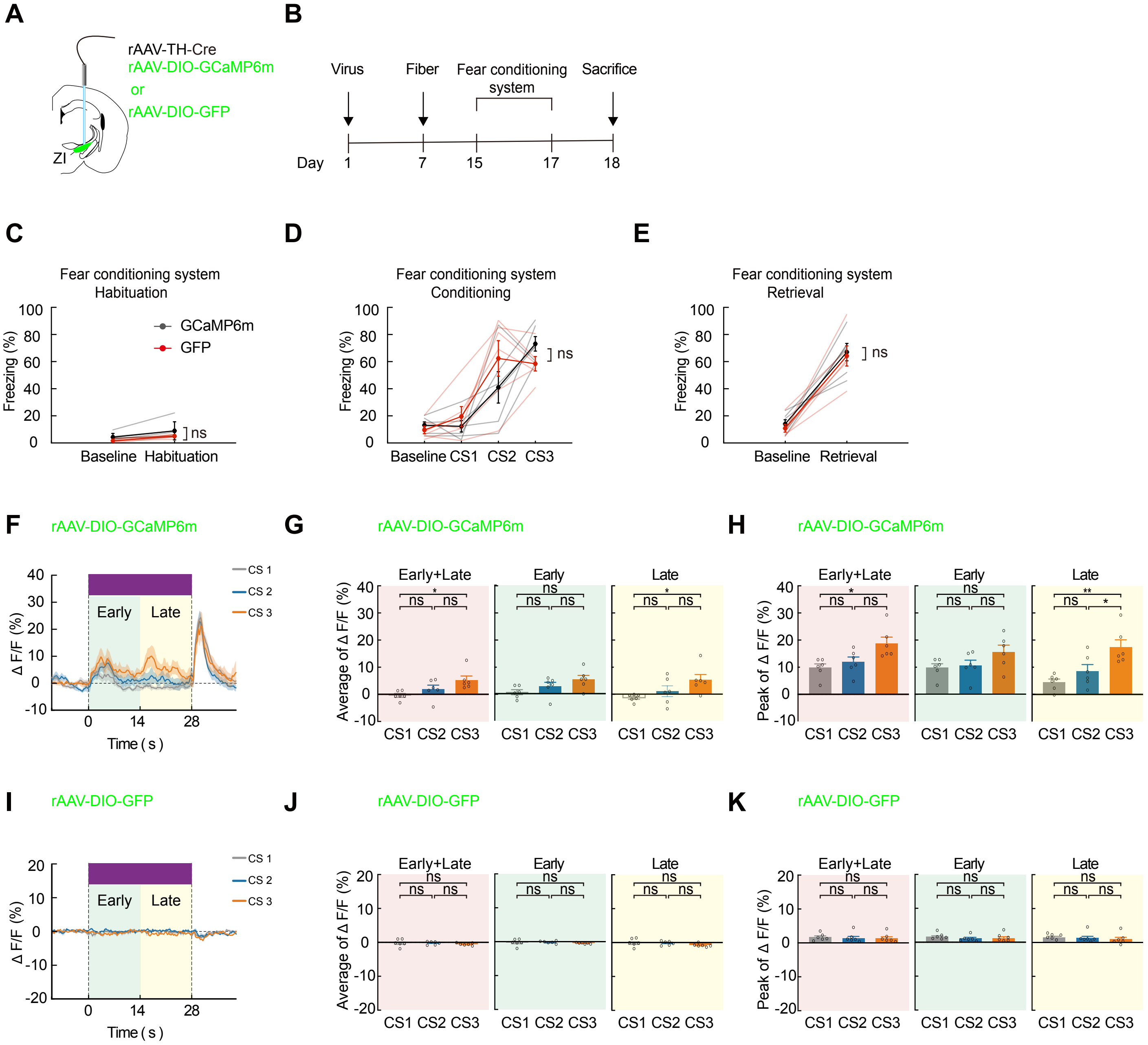

Supplementary Figure 3 | Fluorescence changes from ZI TH+ neurons expressing GCaMP6m or GFP during FCS respectively.

(A) Schematic representation of the virus injection and optic fiber implantation. (B) Experimental protocol timeline. (C) Freezing behavior of GCaMP6m and GFP mice on habituation day. (D) Freezing behavior of GCaMP6m and GFP mice on conditioning day. (E) Freezing behavior of GCaMP6m and GFP mice on retrieval day. (F) Mean fluorescence aligned to CSs delivery of GCaMP6m mice. (G)Comparison of average fluorescence in GCaMP6m mice. (H)Comparison of peak fluorescence in GCaMP6m mice. (I) Mean fluorescence aligned to CSs delivery of GFP mice. (J)Comparison of average fluorescence in GFP mice. (K)Comparison of peak fluorescence in GFP mice.

A

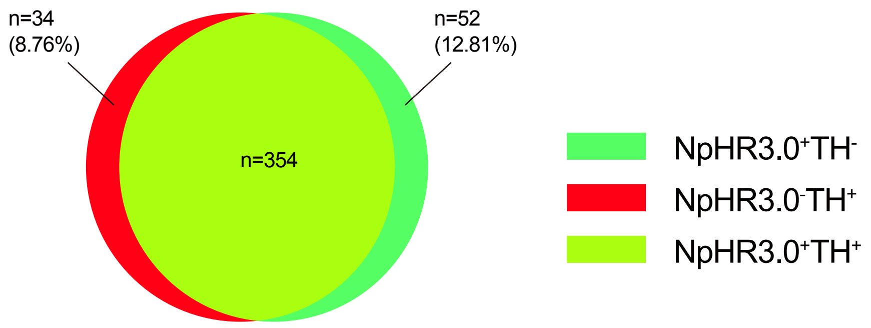

Supplementary Figure 4 | The quantitative analysis of Venn diagram shows the co-expression level of mCherry with TH in ZI.

(A) The quantitative analysis of Venn diagram shows the co-expression level of mCherry with TH in ZI (3 sections per mouse from 3 mice).

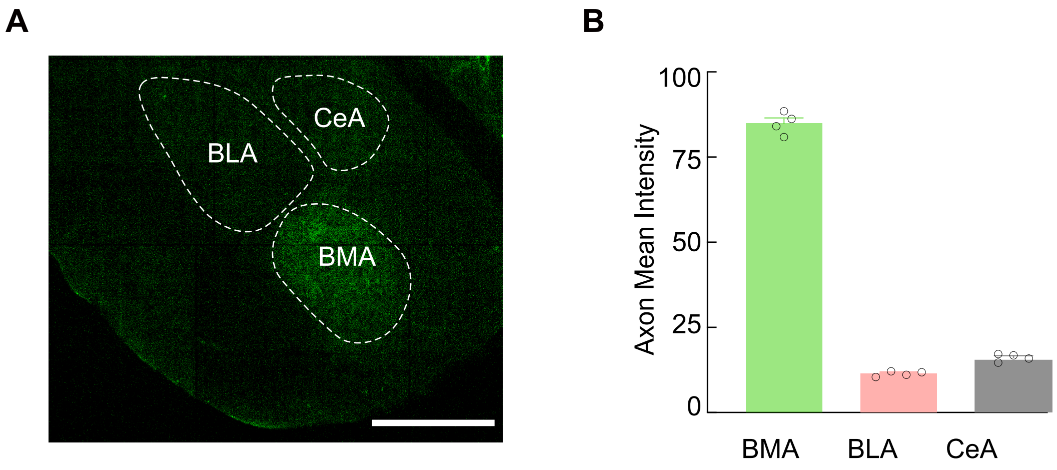

Supplementary Figure 5 | Proportion of ZI-TH+ axons in BMA, BLA and CeA.

(A) ZI-TH+ axons in amygdala. Scale bar, 500μm. (B) Proportion of ZI-TH+ axons in BMA, BLA and CeA.

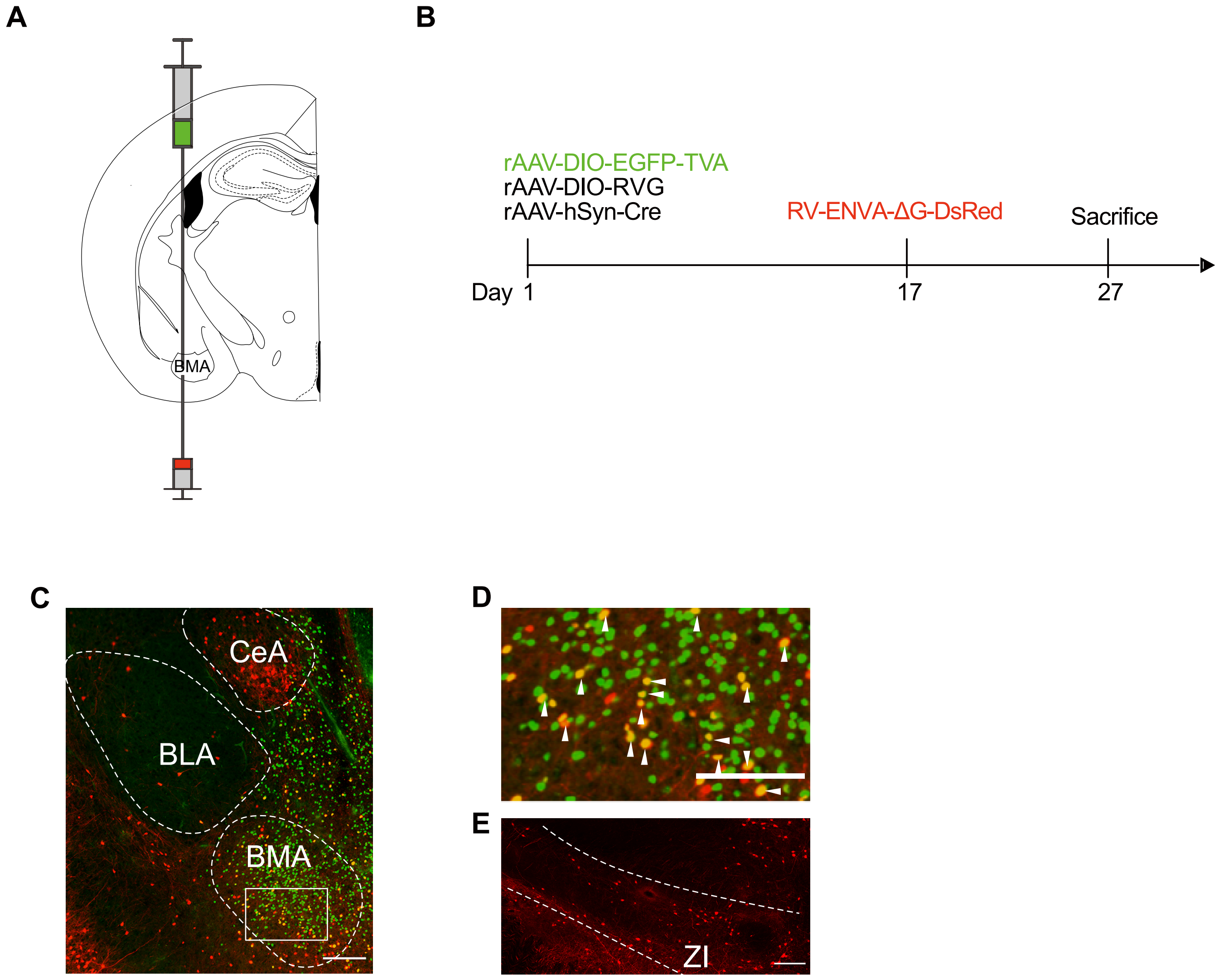

Supplementary Figure 6 | Reconfirm the ZI–BMA circuit by retrograde tracing.

(A) Schematic coronal sections showing virus injection site in the BMA. (B) Experimental scheme of retrograde tracing. (C) Representative fluorescent images of starter cells (yellow) in BMA, which co-infected by rAAV2/9-hSyn-Cre, rAAV2/9-DIO-RVG, rAAV2/9-DIO-TVA-EGFP (green), and RV-ENVA-ΔG-DsRed (red). Scale bar, 200 μm. (D) Higher magnification of BMA area. Scale bars, 200 μm. (E) DsRed-labeled neurons in the ZI traced from the BMA neurons. Scale bars, 200 μm.
